# Children's Attentional Processing of Mother and Proximity Seeking

**DOI:** 10.1371/journal.pone.0124038

**Published:** 2015-04-30

**Authors:** Guy Bosmans, Caroline Braet, Joke Heylen, Rudi De Raedt

**Affiliations:** 1 Parenting and Special Education Research Group, University of Leuven, Leuven, Belgium; 2 Department of Developmental, Personality, and Social Psychology, Ghent University, Ghent, Belgium; 3 Department of Experimental Clinical and Health Psychology, Ghent University, Ghent, Belgium; Utrecht University, NETHERLANDS

## Abstract

Attachment expectations regarding the availability of mother as a source for support are supposed to influence distressed children’s support seeking behavior. Because research is needed to better understand the mechanisms related to support seeking behavior, this study tested the hypothesis that the cognitive processing of mother-related information is linked to proximity and support seeking behavior. Uncertainty in maternal support has been shown to be characterized by a biased attentional encoding of mother, reducing the breadth of children’s attentional field around her. We investigated whether this attentional bias is related to how long distressed children wait before seeking their mother’s proximity. Thirty-three children (9-11 years) participated in this study that consisted of experimental tasks to measure attentional breadth and to observe proximity seeking behavior and of questionnaires to measure confidence in maternal support and experienced distress. Results suggested that distressed children with a more narrow attentional field around their mother wait longer to seek her proximity. Key Message: These findings provide a first support for the hypothesis that the attentional processing of mother is related to children’s attachment behavior.

## Introduction

The quality of parent-child relationships has an effect on distressed children’s attempts to seek and maintain maternal proximity and support [1]. Attachment theory explains this effect by suggesting that experiences with parental care and support become internalized into attachment representations that guide children’s subsequent support seeking behavior [2]. Through the years, attachment research has provided an impressive insight in the importance of attachment for many aspects of child development. However, the crucial question regarding how attachment representations influence proximity and support seeking behavior has long been understudied and little understood. Nevertheless, better understanding these underlying processes is necessary to provide a clearer theoretical framework to explain how parent-child interactions influence child behavior and its development across the lifespan [3]. As lack of proximity and support seeking is a fundamental transdiagnostic risk factor for childhood psychopathology [4], a better understanding of the mechanisms explaining the influence of attachment representations on proximity and support seeking behavior could be fundamental to develop adequate treatment strategies.

It has been proposed that attachment representations are characterized by expectations regarding the attachment figure as a source for support [5]. These attachment-related expectations are presumably reflected in automatic biases in the cognitive processing of attachment-related information, thus organizing attachment behavior in response to distress [6, 7]. The implication is that biases in the processing of attachment-related information might be an essential factor in understanding the organization of attachment behavior. Thus far, the association between attachment-related information processing biases and support seeking behavior has never been tested. Interestingly, recent middle childhood research demonstrated that children’s explicit uncertainty regarding maternal support is characterized by an enhanced attentional focus on mother [8, 9]. As this attachment-related attentional bias might be important to understand the dynamics that influence attachment behavior, the current study aimed to investigate whether this attentional bias is indeed related to distressed children’s inclination to seek their mother’s proximity.

### Confidence in Maternal Support and the Attentional Processing of Mother

Ainsworth [10] conceptualized attachment-related expectations in terms of a child's certainty or *confidence* regarding a primary caregiver's availability, responsiveness, and competence to provide assistance, safety and comfort. Consequently, confident children are supposed to seek parental proximity and support when experiencing distress. This behavior has been named safe haven behavior [11]. Inversely, children who are *uncertain* about parental support are supposed to be less prone to seek parental proximity and support when experiencing distress. As children grow older, confidence in parental support increasingly becomes a crucial facilitator of adaptive development. Instead, uncertainty increasingly puts development at risk, because, for example, reduced support seeking leads to less adequately regulated distress and the development of maladaptive coping styles [12]. Indeed, longitudinal research suggested that the impact of confidence and uncertainty is especially noticeable towards the end of middle childhood [13]. These and similar findings called for research that unravels basic characteristics of attachment-related expectations and safe haven behavior in this age-group.

Cognitive research demonstrated that expectations are characterized by an automatically biased processing of information that is relevant for the content of that expectation. Next to memory [14], and interpretation biases [15], biases in the attentional processing of expectation-relevant information are an important characteristic of expectations because these biases influence all subsequent aspects of information processing and, therefore, are crucial to understand behavioral outcomes [16]. Automatic attentional biases occur outside of individuals’ strategic control, and modulate individuals’ ease to direct their attention towards expectation-relevant information [17]. These biases explain stability of expectations [18] and have an important influence on behavior [19].

Initial research on attachment and attention in children found attachment to be linked with gaze direction [20] and eye movements [21] towards pictorial attachment information. No effect of attachment was found on vulnerability to get distracted from looking at positive and negative scenes during puppet play [22]. Although two of these three studies provided first evidence for a link between attachment and attention, their paradigms measured children’s strategic decisions regarding which information to attend. Consequently, these paradigms did not identify the automatic attention processes that are assumed to determine behavior [23].

A clear example of automatic attentional bias is the breadth of the attentional field around expectation-relevant stimuli presented centrally in the attentional focus [24]. If these stimuli are relevant in light of specific expectations, attentional narrowing occurs [25]. Attentional narrowing refers to “tunnel vision” [26] and means that expectations can reduce the ability to encode information that appears peripheral instead of central to the attentional focus. It is noteworthy that the breadth of attention has a strong impact on the amount of information that is perceived in the environment and consequently on emotions and behavior [27].

**Fig 1:**
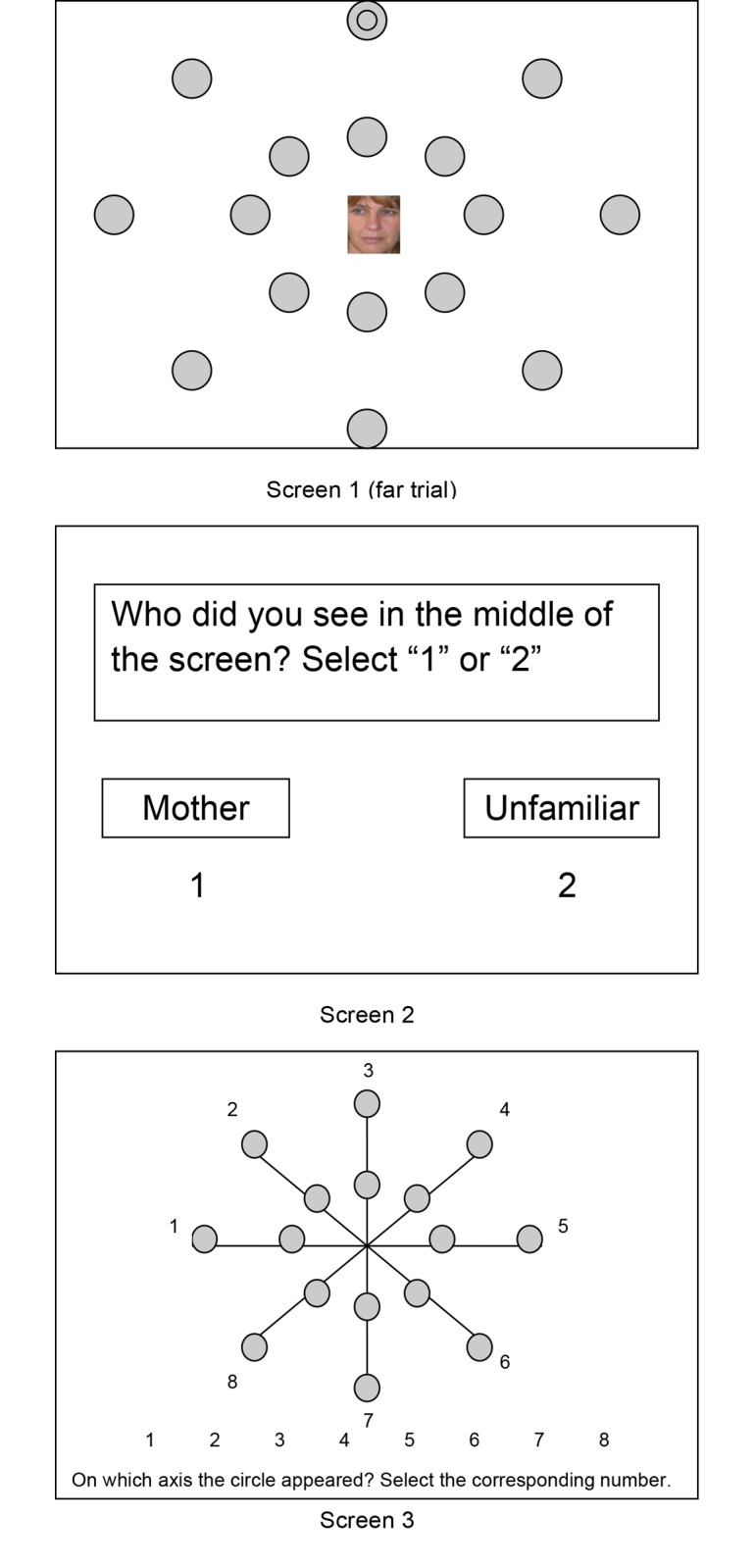
Stimulus Presentation of the Attentional Breadth Task

Because previous research on attentional biases suggested that attention is mainly drawn to stimuli that are object of worry and rumination [24], and because observation of parent-infant interactions suggested that insecurely attached infants overly monitor the mother’s presence at the expense of their own exploration [1], Bosmans et al. [9] tested the hypothesis that a more narrow attentional field around their mother reflects a lack of confidence in maternal availability. To test this hypothesis, they designed a computerized task consisting of trials during which either a picture of children’s own mother or a picture of an unfamiliar woman was presented centrally in their attentional focus during 34 ms. Together with the central stimulus, a target stimulus was presented close by or far from the picture (see Fig 1). Children were asked to correctly identify the location of the target stimulus. Comparing the number of correct responses close by versus far from the central picture allowed identifying the breadth of the attentional field around the central picture (further called the Attentional Narrowing Index, ANI). To investigate the extent to which lack of confidence in maternal availability decreases children’s attentional field around female faces when they process their mother, ANIs around two types of stimuli were measured: pictures of unfamiliar women and pictures of children’s own mother. The difference between the ANIs of both picture types (further referred to as difference in ANI, or ΔANI), reflects the extent to which children have a more narrow attentional field around mother compared to unfamiliar women. This index was then used to investigate whether (lack of) confidence in maternal availability was linked with the breadth of children’s attentional field around mother.

In a first study with 40 children (ages 9–12), Bosmans et al. [9] found that a more narrow attentional field around mother compared to unfamiliar women was related to children’s self-reported uncertainty regarding maternal availability (measured with the Trust subscale of the People In My Life Questionnaire; [28]). This effect could not be explained by children’s trait anxiety. This finding was in line with research on other attachment-related attentional biases [8, 29] and seemed to reflect uncertain children’s inability to stop seeking confirmation of mother’s availability. Moreover, the effect remained significant in a larger sample of 138 children and in a sample of children referred to a residential child psychiatric unit [30]. Most importantly, Bosmans et al. [30] found that children with less self-reported confidence in maternal support in mother and a more narrow attentional field around her showed higher levels of psychopathology. As psychopathology is, amongst others, linked with lack of support seeking behavior [31, 32], the current study aimed to investigate whether the attentional narrowing effect is linked with actual attachment behavior (i.e., social support seeking).

How this attentional narrowing effect might be related to attachment behavior remains an empirical question and is the main focus of the current study. On the one hand, one could argue that children with a more narrow attentional field around mother might constantly seek her proximity in an attempt to seek reassurance. This should translate in enhanced proximity and support seeking. On the other hand, Bowlby [11] suggested that less confident children are less inclined to seek parental proximity and support when distressed. This should translate in delayed proximity and support seeking when children have a decreased attentional field around mother. To reconcile both theoretical stances, it seems reasonable to assume that behavior related to a decreased attentional field around mother might depend on whether or not children are distressed. In the absence of distress, an increased attentional focus might reflect children’s continuously checking mother’s availability and, therefore, could be related to faster proximity seeking. When distressed, the same children’s tendency to ruminate about mother’s availability might have as effect that it takes them more time before they decide to actually seek her proximity and support. As a consequence, a more narrow attentional field around mother might be related to delayed proximity and support seeking in distressed children.

### The Study of Attachment Behavior in Middle Childhood

Behavior reflecting attachment-related expectations has been investigated most thoroughly in infants and young children with the Strange Situation Procedure [1]. The Strange Situation employs a series of brief child-mother separations and reunions to activate the attachment behavioral system and to elicit proximity and contact behaviors. The observed Strange Situation behaviors have been validated against mother-child interactions at home [1], suggesting that experimental observation procedures can elicit attachment-relevant behavior.

Distressed insecurely attached infants respond to the Strange Situation in a more avoidant or a more resistant manner [1]. Critically, these avoidant and resistant behaviors are interpreted as both reflecting lack of confidence in maternal support [1]. Although these avoidant and resistant behaviors are often discussed in trait-like terms, research convincingly shows that these behavioral response patterns should be seen as test-behaviors that are specific to the Strange Situation [33]. For example, less crying during separation is an indicator of avoidant attachment behavior during the Strange Situation, while avoidant infants cry substantially more at home [1]. Also, following up these infants into preschool-age, both insecure attachment classifications were observed to be equally dependent of attachment figures [34]. In the same vein, during an observation study in adulthood, all insecure attachment classifications predicted reduced observed safe haven behavior [35]. These studies suggested that attachment behaviors can be observed during experimental observation procedures, but that the observed behaviors should not be seen as cross-situational stable patterns, but as situation-specific signs of more or less confidence in the attachment figure’s support. Thus, attachment researchers view behavior in the Strange Situation as signs of underlying attachment problems, not as examples of the kind of behavior that will be seen at home.

Beyond infancy, the Strange Situation procedure has been used less to measure attachment behavior. In older preschool children several studies suggested that insecurely attached children wait longer to seek maternal proximity and support [36, 37, 38, 39, 40]. Even less research has successfully observed attachment-related behavior in middle childhood. In fact, in a recent review [12] only a very limited number of studies were described. Moreover, these studies focused on children in the transition from preschool to middle childhood (6–7 years old). To observe these children’s attachment behavior, the Strange Situation procedure was adapted with separation phases lasting approximately one hour [31, 38, 32]. Overall, the results of these studies suggested that, also in the preschool period and in middle childhood, less securely attached children show reduced safe haven behavior. However, young children differ substantially from older children in middle childhood (10–13 years old) [41]. For example, cognitive maturation in older children would require even longer and ethically not justifiable separations before the separation becomes distressing [42, 43]. Moreover, as attachment behavior becomes less visible [44], the Strange Situation procedure can no longer be used in later middle childhood. Therefore, answering the current study’s research question required developing an alternative procedure to elicit attachment-related behavior. Following the Strange Situation procedure rationale and given the abovementioned reasons why the Strange Situation procedure is no longer appropriate in older children, this new procedure needed to (1) induce distress relevant to activate the need for maternal proximity and support, and (2) elicit observable attachment behavior.

Firstly, to select an age-relevant source of distress, this new procedure was based on middle childhood theory and research. Theoretically, Mayseless [44] assumed that, at this age, the attachment system gets activated when children experience hurt pride. This implies that hurt pride should induce a need for maternal proximity and support. Research further suggested that tasks involving social comparison with peers become increasingly distressing in this age-group due to feelings of hurt pride, but only in the absence of parental proximity and support [45]. Therefore, we argued that this source of distress might activate the need for maternal proximity and support.

Secondly, to identify observable attachment behavior, we argued that how long distressed children wait to seek their mother’s proximity could be a sign of more or less confidence in her support. Based on Bowlby’s [11] abovementioned assumption that distressed children with less confidence seek less maternal support, and based on Crowell et al.’s [35] observed confirmation of this assumption, we predicted that waiting longer to seek proximity could be a behavioral indicator of lack of confidence in maternal support. Building on these ideas, a new attachment observation procedure was developed in which attachment behavior was conceptualized as the time children waited before ringing a bell to call mother. Children were told that their mother would return immediately after ringing the bell. The procedure consisted of two phases. In a first phase, a difficult puzzle task was administered. To induce age-relevant hurt pride-related distress, children were told that all their peers easily solved the puzzle before. Because the distress that activates attachment behavior in the strange situation procedure refers to children’s frustrated need to be close to mother [1], we aimed to induce a similar need in a second phase of the observation procedure. In the latter phase, mother did not come after ringing for her to see how long children waited to call her a second time.

### The Present Study

In the present study we tested the hypothesis that distressed children’s decreased attentional field around mother, which is associated with insecure attachment, is linked with a delay in calling mother. For Phase 1, this leads to the prediction that distressed children with a more narrow attentional field wait longer to call mother a first time. As distress should be most pronounced when children actually fail to solve the puzzle, analyses for Phase 1 were performed separately for children who did and who did not solve the puzzle. For Phase 2, one could argue that more securely attached children might wait longer before calling mother a second time, because they are more confident in her return. However, in congruence with the Phase 1 hypothesis, we predicted that children with a more narrow attentional field would be least prone to call mother a second time, but only if her delayed return induces distress. Therefore, distress while waiting for mother was used as a moderator in the analyses for Phase 2. As both phases’ waiting times could be related to other cognitive and personality features such as, impulsivity, patience, and task persistence, we added a self-report measure of confidence in maternal support to investigate whether waiting time also relates to that alternative attachment-relevant measure and to investigate whether the shared variance between attentional narrowing around mother and waiting time indeed relates to (lack of) confidence. Finally, attentional biases in general and the breadth of the attentional field in specific are known to be strongly linked to anxiety [26, 46]. Therefore, trait anxiety was measured as a control variable.

In summary, the current study aimed to investigate the relationship between the attentional processing of mother and distressed children’s proximity seeking behavior. Firstly, the hypothesis was tested that children with a narrow attentional field around mother wait longer to call her after experiencing difficulties solving a supposedly easy puzzle. Secondly, the hypothesis was tested that children with a narrow attentional field wait longer to call for mother a second time if the delay in mother’s return further induced distress. Thirdly, the hypothesis was tested that confidence in maternal support is associated with waiting time and explains associations between the attentional narrowing effect and proximity seeking behaviors.

## Method

### Participants

In this study, 33 children participated (17 boys, 16 girls) with ages ranging from 9–11 years (*M* = 10.27; *SD* = .63). Of these children, 84% still lived together with both biological parents. The remaining children lived most frequently together with mother as main caregiver. All mothers were Caucasian and reported they had been the primary attachment figure during the first three years of their children’s lives. Regarding parental highest level of education, 3% of the mothers had an elementary school degree, 33.3% had a high school degree, 24.2% had a post-high school technical training or a technical bachelor degree, and 39.4% had a master’s degree. Furthermore, 33.4% of the fathers had a high school degree, 30.3% had a post-high school technical training or a technical bachelor degree, and 36.4% had a master’s degree.

### Measures

#### The Attentional Narrowing Effect

was measured using the Attentional Breadth Task (ABT), which was based on the design developed by Ball, Beard, Roenker, Miller, and Griggs [47]. Participants were seated in front of a 19” CRT-computer screen, at a distance of exactly 27 cm from the screen using a chin rest to ensure accurate positioning and a computer mouse for answering. At each trial (see Fig 1 for an example of one trial), in the center of the screen a picture appeared (3 cm wide by 4 cm high). Simultaneously with the presentation of the central picture (see Fig 1), 16 gray dots with a diameter of 2 cm appeared at 4.5 cm from the central picture (close trials at 10° of the visual angle) and at 11.2 cm from the central picture (far trials at 25°) appeared during 34 ms. The grey dots were arranged in pairs of two (one close and one far dot, situated on one of eight imperceptible axes that came together in the central point were the central picture was shown). Together with the dots, in one of these dots a smaller black circle with a diameter of 1.3 cm appeared either in one of the close or in one of the far dots. This black circle was the target stimulus that participants had to identify. After each trial a screen appeared with the question which picture they had seen (mother or unfamiliar woman). The amount of correct responses on this question shows whether the participants were looking at the center of the screen. Then a second screen appeared showing the axes with the question on which of eight axes the target stimulus was located.

The pictures were divided into two categories: For the unfamiliar category, ten pictures were taken of 10 different Caucasian women unfamiliar to the participants. At the beginning of the experiment, ten different pictures of the participating children’s mother were taken, focusing on the mother’s face, and avoiding bright colors in the pictures. The mother was asked to show a neutral face, as much as possible without showing her teeth to avoid salience effects. The experimenter took the mother’s pictures with a digital photo camera. Four categories of trials were presented with two picture types (mother versus unfamiliar women), and two distances (target stimulus presented close or far from the central picture). Thus, the categories were (1) mother close, (2) mother far, (3) unfamiliar close, (4) unfamiliar far. Each category contained 16 trials. The trials were presented in two blocks of 64 trials each, separated by a short break.

For all analyses, only the trials with correctly identified central pictures were used. This ensures that attention was focused to the middle of the screen. The proportion of correctly identified targets on trials with correctly identified pictures served as the main dependent variable. An Attentional Narrowing Index (ANI) was calculated by subtracting the proportion of correctly identified targets in the far trials from the proportion of correctly identified targets in the close trails. The ANI was calculated for mothers (ANImother) and unfamiliar women (ANIunfamiliar). Finally, a ΔANI-effect was calculated subtracting ANIunfamiliar from ANImother, which expresses the extent to which the decrease in attentional breadth is stronger around mother compared to unfamiliar women. ΔANI is the index that was used in all analyses to investigate the attachment-related attentional narrowing effect.

#### Proximity Seeking Behavior

First, children were asked to complete a puzzle (taken from the Rush Hour Traffic Jam Puzzle, which was used before to induce distress in middle childhood by Eldar, Ricon and Bar-Haim [48]. After an easy example was solved together with the experimenter, children were given a second puzzle to solve. Although the second puzzle appeared to be as easy as the example puzzle, the level of difficulty was substantially higher. Nevertheless, the children were told that it was an easy task, that all their peers had been able to solve it, and that it was important to perform well. We assumed that children’s experience that the supposedly easy puzzle was hard to solve could serve as a source of hurt pride, thus activating the need for maternal support. Then, the experimenter left the room after giving the child a wireless doorbell and after instructing that if the task was finished or when (s)he wanted to stop that (s)he had to ring the bell and that mother would come immediately to get her/him. To make sure that the children would know that the doorbell worked, the button had a light that illuminated after pushing the button and the doorbell was tried out once so children could hear that the wireless bell functioned. The time before the child pressed the bell is the first dependent variable (Bell 1, expressed in minutes). Meanwhile, mothers were asked to wait for the child to ring a second time (with a maximum waiting time of 10 minutes) before going to their child. We assumed that this would induce distress comparable to infants’ distress in the strange situation due to their frustrated need of being close to mother. The time between the first and the second bell was the second dependent variable (Waiting Time, expressed in minutes).

#### Manipulation check

Three items were constructed to measure whether the manipulation worked. (1) Children were asked whether they believed the experimenter’s suggestion that all their peers had been able to solve the puzzle. (2) Children were asked how nervous they got experiencing that the puzzle was much more difficult to solve than they had expected. (3) Children were asked how well they already knew the game. All items were scored on a Likert-scale, ranging from 0 (not at all) to 4 (very much).

#### Distress while waiting for mother after Bell 1

Children were asked to indicate “How distressed did you feel when your mom did not show up?” on a Likert-scale, ranging from 0 (not at all) to 4 (very much).

#### Confidence in maternal support

was estimated with the Trust subscale of the People In My Life Questionnaire [28] which is a child-friendly version of the Inventory of Parent and Peer Attachment [49]. This questionnaire is widely used to investigate Trust in the attachment figures’ support, Communication about distress, and Alienation from the attachment figure [50, 51, 52]. For the current study, only the items of the Trust-scale focusing on the relationship with mother were used. The Trust scale is conceptualized as the positive affective/cognitive experiences of confidence in the accessibility and responsiveness of attachment figures (10 items, e.g. “I can count on my mother to help me when I have a problem”). Children responded on a 4-point Likert-scale ranging from 1 (“*almost never true*”) to 4 (“*almost always true*”). The Trust scale was reliable in the current sample (*α* = .86).

#### Trait Anxiety

was measured using the Trait Anxiety subscale of the State-Trait Anxiety Inventory for Children [53], translated into Dutch by Bakker, van Wieringen, van der Ploeg and Spielberger [54], which was administered to the children. Trait Anxiety was previously reliably measured in a Dutch-speaking population of preadolescence [55]. In the current sample, *α* = .74.

### Procedure

Using a flyer distributed in the classrooms of the fifth and sixth grade of elementary schools, we invited volunteering children and their parents to the laboratory for a study on the relationship between children and their mother in return for two access tickets to the movie theatre. Two hundred flyers were distributed, informing parents about the content of the study and asking their approval to participate. Thirty-seven parents who received a letter gave their written informed consent. All children who participated, signed an additional assent form after being personally informed about the content and the methodology of the study and about their right to refuse participation. The first four children participated in a pilot version of the task, which was needed to fine-tune the procedure. The remaining 33 children all followed the same procedure. First, ten pictures were taken from the mother. While the child completed the questionnaires, the photos were integrated into the ABT. After the ABT, the children were presented with the difficult puzzle and left alone with the doorbell. After the mother entered the room, the experimenter joined in and gave the additional Distress and Manipulation Check items. Using a one-sample *t*-test (comparing scores to 0) we found that children on average believed the experimenter (*t*(32) = 17.54, *p* < .001; *M* = 3.24, *SD* = 1.06) and that the experience that the puzzle was more difficult than expected was distressing (*t*(32) = 8.24, *p* < .001; *M* = 1.76, *SD* = 1.23). Moreover, game knowledge was not correlated with the dependent variables. After the experiment, children were completely debriefed, and informed about the misleading information. Only after it was clear that children were relaxed and experienced no side effects, the lab visit was finished. Finally, to account for possible long-term effects, parents received contact information of the first author, a licensed child therapist, and contact information of the local outpatient mental health service. The entire procedure was approved by the Ghent University ethics committee. Written informed consent was obtained from both parents and children. The informed consent protocol and the informed consent letters for both parents and children were all reviewed and approved by the Ghent University ethics committee. The individual in Fig 1 has given written informed consent (as outlined in the PLOS consent form) to publish these case details

## Results

### Preliminary Analyses

Only 1% of the data was missing, but the Trust score of four children was missing due to skipped items. As these missing data were completely at random (Little’s MCAR test was not significant: χ²(112) = 13.87, *p* = 1) the expectation maximization (EM) method was used to estimate the missing scores (although also without EM, the same results were found). There were no outliers. Gender was not correlated with the dependent variables. Regarding the ABT, a 2 (Distance: Close vs Far trials) x 2 (Picture: Mother vs Unfamiliar Women) Repeated Measures ANOVA revealed a significant distance effect, *F*(1, 32) = 247.30, *p* < .001, confirming enhanced task difficulty in far trials. Trait Anxiety was not correlated with ΔANI (*r* = -.08, *ns*), Trust (-.27, *ns*), Bell 1 (*r* = -.02, *ns*), nor Waiting Time (*r* = .07, *ns*) and thus did not affect the reported analyses. Finally, Bell 1 and Waiting Time were not significantly correlated (*r* = .04, *ns*) and whether or not children solved the puzzle did not affect Waiting Time (*F*(1, 31) = 2.23, *ns*). This suggests that the outcomes of Phases 1 and 2 are independent and that Waiting Time during Phase 2 was not affected by whether or not children solved the puzzle during Phase 1. Table 1 displays descriptive statistics for each key measure across the entire sample.

**Table 1:** Descriptive statistics.

	M	SD	Minimum	Maximum
Trust	36.56	2.58	30.00	40.00
ΔANI	-.02	.11	-.27	.24
Bell 1	11.45	6.98	2.00	32.00
Waiting Time	2.67	2.46	0.00	9.00

### Attentional Breadth and Proximity Seeking during the Problem Solving Task

On average, children rang a first time after 11.46 minutes (*SD* = 6.98). Overall, time to Bell 1 tended to correlate with ΔANI (*r* = .32, *p* = .07). The puzzle was correctly solved by 17 children (51.5%). To investigate whether solving the puzzle reduced the amount of distress experienced, we carried out an independent-samples *t*-test with solving the puzzle (1, 0) as grouping variable and children’s answer to the item “how nervous did you get experiencing that the puzzle was much harder to solve than you had expected” as test variable. However, solving the puzzle or not did not appear to change the level of distress induced by the puzzle (*t*(31) = 1.41, *ns*). Nevertheless, the time children waited to call mother after successfully solving the task was function of the time needed to find the solution instead of hurt pride. By consequence, Bell 1 in these children should be less directly related to the need to be close to mother. Therefore, associations with ΔANI should mainly be significant after children failed to solve the puzzle. Table 2 confirms that only in the latter group Bell 1 was strongly and significantly correlated with ΔANI explaining 35% of the variance in Bell 1. After controlling for Trait Anxiety, the correlation between ΔANI and Bell 1 was marginally significant for the entire group (*r* = .32, *p* = .08), and significant for the group that failed to solve the puzzle (*r* = .59, *p* < .05). This correlation did not differ significantly from the group that did manage to solve the puzzle (*z* = -1.18, *ns*).

**Table 2:** Correlations between Attachment Measures and Attachment Behavior.

	Trust	ΔANI
Time before Bell 1		
Entire Sample	-.53* **	.32^†^
Solution found	-.46^†^	.22
Solution not found	-.71**	.59*

^†^ *p* = .07;

* *p* < .05;

** *p* < .01

### Attentional Breadth and Proximity Seeking while Waiting for Mother

**Fig 2:**
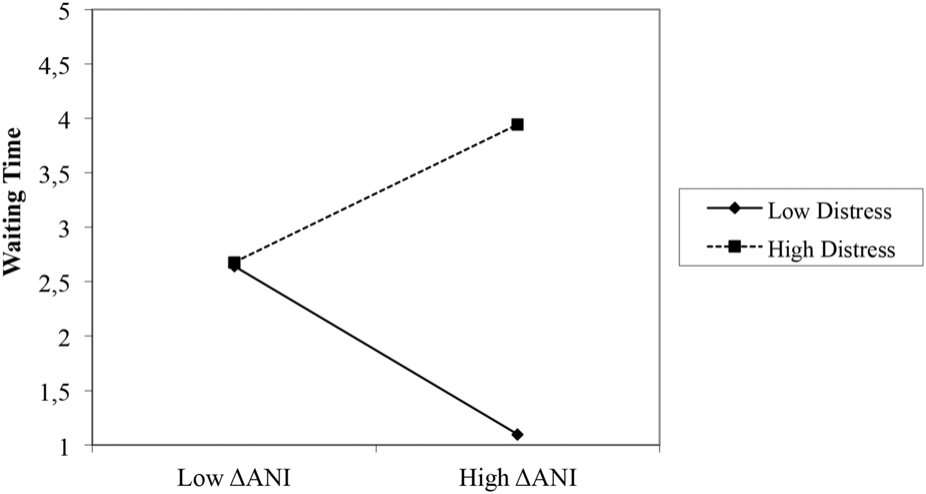
Interaction between Distress and ΔANI predicting Waiting Time.

All children rang a second time within ten minutes (range 0–9 minutes, *M* = 2.67, *SD* = 2.46). Waiting Time was not correlated with ΔANI (*r* = .15, *ns*). The interactions between ΔANI and Distress on Waiting Time were investigated using Hierarchical Multiple Regression Analysis [56]. A significant interaction was found between Distress and ΔANI (see Table 3) explaining 26% of the variance in Waiting Time. The significance of the unstandardized regression weights of the slopes was calculated using Hayes and Matthes’ SPSS macro [57]. None of the slopes reached significance. However, children with higher ΔANI scores waited significantly longer to call mother a second time when they were more distressed, *t*(31) = 3.04, *p* < .01 (see Fig 2). The interaction effect remained at least marginally significant in spite of controlling for Bell 1 (*β* = .39, *p* < .05), whether or not the children found the solution to the puzzle (*β* = .33, *p* = .06), and Trait Anxiety (*β* = .38, *p* < .05).

**Table 3:** Interaction between Distress and ΔANI Predicting Waiting Time.

	*R^2^Δ*	*F Δ*	*df*	*β*
Step 1	.15	2.73	2, 30	
ΔANI				-.03
Distress				-.29^†^
Step 3	.11	4.25	1, 29	
ΔANI x Distress				-.37*

Note: All reported *β* are values at Step 3 of analysis

^†^ *p* < .1;

* p < .05;

** *p* < .01;

*** *p* < .001

### Trust, Attentional Breadth, and Proximity Seeking

Trust was significantly correlated with ΔANI (*r* = -.39, *p* < .05) and with Time to Bell 1 (*r* = -.53, *p* < .001)^2^. In a Multiple Regression Analysis (MRA) predicting Bell 1, the marginally significant effect of ΔANI was reduced to non-significance (*β* = .14, *ns*) after taking into account the effect of Trust (*β* = -.48, *p* < .01).

**Fig 3:**
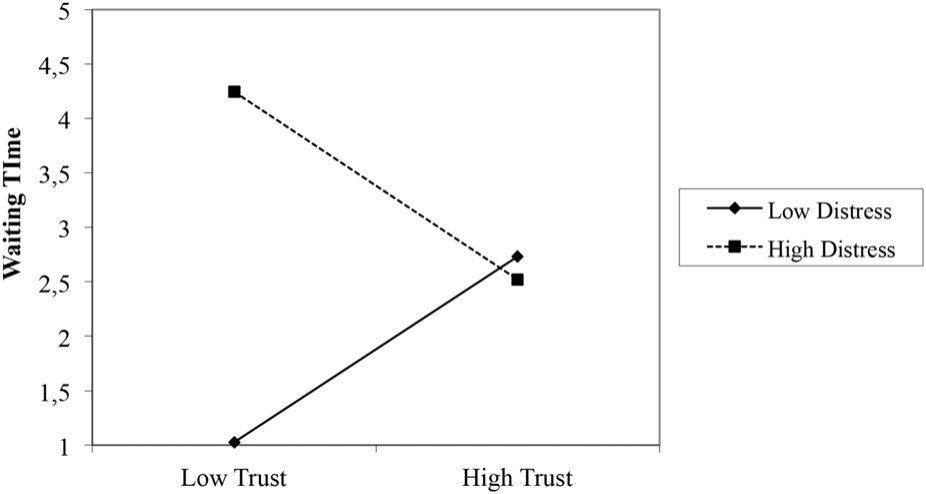
Interaction between Distress and Trust predicting Waiting Time.

Waiting Time was not correlated with Trust. A significant interaction was found between Distress and Trust (see Table 4) explaining 29% of the variance in Waiting Time. None of the slopes reached significance. However, Fig 3 demonstrates that children with lower Trust scores waited significantly longer to call mother a second time when they were more distressed, *t*(31) = 3.34, *p* < .01. In an MRA predicting Waiting Time, the interaction effect with ΔANI was reduced to non-significance (*β* = .23, *ns*) after taking into account the interaction effect with Trust (*β* = -.21, *ns*).

**Table 4:** Interaction between Distress and Trust Predicting Waiting Time.

	*R^2^Δ*	*F Δ*	*Df*	*β*
Step 1	.16	2.85	2, 30	
Trust				-.00
Distress				-.31^†^
Step 3	.13	5.28	1, 29	
Trust x Distress				-.39*

Note: All reported *β* are values at Step 3 of analysis

^†^ *p* < .1;

* p < .05;

** *p* < .01;

*** *p* < .001

## Discussion

The current study investigated whether a more narrow attentional field around mother is related to distressed children’s delayed proximity seeking. For this purpose, children were exposed to mild distress in order to elicit attachment-related proximity seeking behavior. Three hypotheses were tested. First, we tested whether children with a narrow attentional field around mother wait longer to call her after experiencing difficulties solving a supposedly easy puzzle. Second, we tested whether these children wait longer to call for mother a second time. Third, we tested whether confidence in maternal support is associated with waiting time and explains associations between the attentional narrowing effect and proximity seeking behaviors. Results largely supported our central hypotheses.

Before discussing the results in detail, it was important to evaluate the novel procedure we developed to elicit attachment-relevant behavior in middle childhood. Confirming this new procedure’s basic assumption and in line with previous research [45], performing worse than peers in solving the puzzle task induced significant distress. Solving the puzzle or not did not affect the level of distress. This suggests that it is likely that distress was due to the overall comparison with peers and not just to failure to solve the puzzle.

The times children waited to call mother a first and a second time were not correlated. Moreover, whether or not children managed to solve the puzzle did not affect waiting time in the second phase of the procedure. This was important because data interpretation would have been complicated if longer time before calling a first time would have been linked with decreased waiting before calling a second time. Such a finding would have suggested that children who tried longer to solve the puzzle were less patient to wait for mother as they already spent much time in the lab. This could have reflected the wish to leave the lab and not the need to reunite with mother. Nevertheless, given the assumption that attachment needs underlie calling behavior in both phases, a positive correlation would not have been unexpected. Because a correlation between the times children waited to call mother in both phases could have been masked by the fact that not all children experienced distress during the second phase, we performed a median split on distress experienced during mother’s unexpectedly delayed return. Although this analysis was rough and ignored relevant individual differences, it was interesting to see that in children who reported more distress while waiting for her return, the correlation between Bell 1 and Waiting Time was significantly positive (*r* = .47, *p* < .05). In sum, all these findings suggested that this new procedure could be useful to investigate the hypothesis that biases in the attentional processing of mother are related to attachment behavior.

The current study’s first hypothesis was tested in Phase 1. Results supported the hypothesis that children with a more narrow attentional field around mother wait longer to call her during the distressing task. This finding suggests that uncertain children with an enhanced attentional focus on mother show delayed proximity seeking behavior in response to distress. This finding seems in line with Mayseless’ [44] assumption that this source of distress activates the attachment system. Interestingly, this effect appeared to be more pronounced when children failed to solve the puzzle. Although the difference between both groups’ attentional breadth-waiting time correlations did not reach significance, this seemed to further support the validity of the new procedure’s basic assumption: only in children who failed to solve the puzzle could the decision to call for mother be driven by their need to seek her proximity for support. The other children called mother at the moment they had finished the task, not when hurt pride due to failing to solve the task raised to the level that their need for mother’s proximity resulted in calling behavior.

The second hypothesis was tested in Phase 2. The significant interaction effect of Distress that was found suggests that level of distress modulated how long children with a more narrow attentional field around mother waited to call for her a second time. More specifically, when mother’s absence induced distress these children waited longer to seek her proximity. This effect was in line with Bowlby’s [2] prediction that distress is an important moderator of attachment behavior. However, the slopes of the interaction effect were not significant, so one cannot conclude that this effect merely replicated the effect found in Phase 1. Nevertheless, waiting time in Phase 2 in children with a narrow attentional field around mother depended significantly on level of distress. This finding is in line with our hypothesis that distressed children with a narrow attentional field around mother are less likely to seek her proximity. Moreover, the moderating effect of distress was in line with existing theory and research findings. Theoretically, Sroufe and Waters [58] argued that attachment is an organizational construct that leads to different behavioral outcomes in different circumstances. Empirically, this effect was in line with the findings of Ainsworth et al. [1] and Sroufe et al. [34] that showed that attachment-related behavior patterns are context dependent. Hence, we argued that also Phase 2 provided some evidence that a more narrow attentional field is related to reduced proximity seeking in distressed children.

The third hypothesis was tested in both Phases. Because this is a novel paradigm, one could argue that waiting behavior could reflect children’s characteristics not related to attachment. It is not impossible that waiting time during both phases could have been due to features such as impulsivity, patience, task perseverance, or even self-esteem and self-confidence. To control for the effect of any confounding variables, we should have included several other measures as control variables in the statistical analyses. However, including these measures was not feasible in light of the already time-consuming and demanding procedure. As a preliminary test to further evaluate whether the effects found in the current study were specifically relevant for attachment, we investigated links with self-reported confidence in maternal support. Importantly, all the effects found with the attentional breadth task could be replicated with this alternative attachment measure. Moreover, the link between the attentional narrowing effect and proximity seeking was fully accounted for by children’s self-reported confidence in mother’s support. This further suggests that the current study provides some first evidence suggesting that attentional processing of mother is involved in children’s attempts to seek maternal support. Nevertheless, given the novelty of the paradigm, more research is needed to further confirm the attachment-relevant nature of the waiting behavior. Also, the current study’s design does not allow testing the underlying theoretical assumption that attention has a causal effect on behavior. To reveal such a causal effect, future research should manipulate attention in order to investigate effects on behavioral change. However, given the lack of good observation paradigms, it was promising that the newly developed paradigm did seem to create the conditions needed to investigate the current study’s research question. Therefore, the current study was important as it provided first evidence suggesting that a link between attachment-related attention and behavior exists and that the direction of effects is in line with previous attachment observation research [35, 36, 37, 38, 39].

In future research, the function of this association needs to be unraveled. Bowlby [2] emphasized the function of attachment and the role of proximity in safety seeking and predator avoidance. The present data are in line with the idea that a less narrow attentional field around mother enhances the ability to seek proximity and support. One possible explanation might be that delay in proximity seeking reflects a cognitive approach-avoidance conflict. Evidence is found that a narrow attentional field around stimuli not only indicates that these stimuli elicit intense avoidance-motivating worry [24], but, at the same time, such stimuli might elicit approach-motivating affect [59]. Such attention-related approach-avoidance conflicts are known to delay behavioral decisions [60]. Translated to the present study, a narrow attentional field around mother might simultaneously reflect uncertain children’s wish to approach mother, but also to avoid mother as she is object of worry. It seems reasonable to assume that this decision process delays proximity seeking responses. Though this explanation is plausible, other explanations should be investigated as well. For example, it could be that confident children need to consider less coping options than uncertain children. While confident children have learned that support seeking is the best option to cope with distress, uncertain children may need to consider several fight- and flight-related options next to proximity seeking. Considering more options implies spending more time before taking action.

Finally, the current study’s approach to measure the breadth of children’s attentional field around mother and self-reported confidence in maternal support did not allow distinguishing patterns of insecure attachment behavior. One could expect different effects for more avoidantly versus more resistant or anxiously insecurely attached children. More specifically, more avoidantly attached children could be most delayed in their proximity and support seeking, while more anxiously attached children could be the fastest to seek maternal proximity and support [61, 62]. However, the current study’s attachment measures could not be used to test this hypothesis. At best, the current findings seem to suggest that there was a general effect of secure versus insecure attachment on the observed attachment behavior. Interestingly, some observation studies showed similar behavior patterns for different attachment styles [1, 35, 34]. In these studies, these findings were interpreted as reflecting avoidant and anxious individuals’ shared characteristic, the hallmark of insecure attachment, namely lack of confidence in maternal support. Consequently, there are some arguments to propose that the time children wait before they seek maternal proximity and support might be similar for avoidantly and anxiously attached children. Future research should test this hypothesis more directly, including a measure of avoidant and anxious attachment. In light of the current discussion, it is not irrelevant that we recently did a middle childhood study (*n* = 98) with an adjusted puzzle task and a self-report measure of avoidant and anxious attachment revealing delayed proximity and support seeking for both insecure attachment dimensions.

### Limitations

This study is a first small yet promising step towards a better understanding of the mechanisms underlying the attachment system. However, more research is required as the current study had some important limitations. Above all, the sample size was small. Even though lower power accentuates the importance of the findings, one cannot rule out that some unforeseen sampling effects might have driven the results. The problem with small samples is that the findings might not accurately reflect the true population values. For example, a substantial proportion of the parents in the current sample had a master’s degree, suggesting that the sample was very selective. Although, it is promising that we found these effects in spite of the homogeneous nature of the sample, it remains a question whether the current effects translate to a more heterogeneous sample. So replication of the presented results in a larger and more representative sample is necessary.

Second, the ABT compares responses to pictures of mother versus unfamiliar women. Although this manipulation is useful to investigate the extent to which children’s evaluation of mother affects their attentional processing of comparable, but neutral social information, it is hard to exclude whether the effects are driven by frequency of presentation (one mother versus 10 unfamiliar women) or by familiarity of the stimulus (and not by attachment). Future ABT research could compare pictures of one mother with pictures of the same unfamiliar woman, but risks to lose ecological validity because then results could be confounded by coincidental features of the unfamiliar woman (e.g., attractiveness or other physical characteristics [9]). Future research could also compare pictures of mother with pictures of other familiar figures (e.g., siblings). However, such approach could mask attachment effects, because some evidence suggests that attachment representations are not specific for one relationship [63]. Finally, it could be an interesting idea to also apply eye-tracking technology to detect true-center-fixation. However, eye-tracking methodology often requires longer presentation times, which affects the direction and meaning of the attachment-related attentional processing effects [64].

Third, although success on the puzzle task had no effect on the level of induced distress, and although the differences in solving the puzzle allowed demonstrating the validity of the current procedure, it would be advisable to design a version of the procedure during which all participants fail to solve the puzzle. The current results suggest that such a design might reveal even clearer links with attachment-relevant variables. The current procedure might be improved by using a different stress inducing task.

Finally, attachment was measured using a self-report measure. It has been argued that self-report is a less valid approach to measure attachment as it over identifies security [65]. Nevertheless, in the same paper, Ainsworth argued that self-reported insecure attachment can be considered more valid and reliable. Moreover, instruments to measure attachment in middle childhood have long been lacking [42]. The last years, it became widely accepted to measure middle childhood attachment using questionnaires [66]. Moreover, research shows that middle childhood attachment questionnaires correlate convincingly with middle childhood attachment interviews and other narrative tasks [67]. Furthermore, in the current study, the attachment measure functioned merely as a control variable, and explained the effect of the attentional bias on proximity seeking behavior. As the attentional bias cannot be strategically controlled, it might be that, at least in this sample, under identification of insecure attachment was less problematic. Nevertheless, future research might benefit from measuring unconscious aspects of attachment representations instead of using questionnaires.

### Theoretical Implications

In spite of these concerns, to evaluate the relevance of the current findings, it is important to keep in mind that correlations between experimental tasks and very specific behaviors are difficult to find [68]. The strength of these associations in a small sample suggests that this attentional bias represents a powerful phenomenon that could be relevant to understand some of the understudied mechanism in the attachment system. The fact that this attentional bias occurs at very short presentation times (34 ms) could mean that some of the processes that organize attachment behavior occur at a pace that makes it hard for children to strategically or consciously modulate attachment behavior.

In an attempt to clarify the theoretical importance of this finding, we would like to give the following example. Imagine a 10 year old child that is being bullied at school. Attachment theory would argue that the best coping strategy for this child would be to seek maternal proximity and support. Indeed, distress will probably activate the attachment system, guiding proximity and support seeking behaviors [1]. However, if the child is uncertain about maternal support, its attention will be automatically restricted to focusing on mother [9]. This attentional bias probably reflects this child’s rumination about mother’s availability [24] and limits this child’s resources to seek maternal proximity. Consequently, this automatic attentional bias might be important to understand why insecure attachment increases the likelihood to develop psychopathology, why attachment is a cross-temporally stable construct, and why attachment is difficult to change through therapy [3]. Clinically, this reasoning suggests that new therapeutic strategies are required in order to change information processing biases. One approach could be the use of Cognitive Bias Modification [69], which aims to alter the content of cognitive schemas by retraining the biases that accompany these schemas. Adding such a CBM component to the traditional treatment of child and adolescent psychopathology might prove to be a very powerful tool to substantially improve treatment effects.

### Conclusion

In summary, this study provided evidence supporting the hypothesis that biases in the attentional processing of mother influence proximity seeking behavior. More specifically, children with a more narrow attentional field around mother waited longer to seek her proximity when feeling distressed. In spite of limitations regarding sample size and the requirement to replicate these findings to better establish the validity of the current study’s middle childhood attachment observation procedure, the findings could prove to be highly relevant and could open the door to a new line of attachment research that bears the promise of better understanding the processes involved in the attachment system.

## References

[1] Ainsworth MDS, Blehar MC, Waters E, Wall S. Patterns of attachment: A psychological study of the strange situation. Hillsdale (NJ): Erlbaum; 1978.

[2] Bowlby J. Attachment. London: Penguin Books; 1969.

[3] Thompson RA. Attachment-related mental representations: Introduction to the special issue. Attach Hum Dev. 2008; 10: 347–358. 10.1080/14616730802461334 19016046

[4] DeKleyen M, Greenberg MT. Attachment and psychopathology in childhood In: Cassidy J, Shaver PR, editors. Handbook of attachment: Theory, research, and clinical applications. New York (NY): Guilford; 2008 p. 93–113.

[5] Bowlby J. Attachment and loss: Vol. 2 Separation. New York (NY): Basic Books; 1973.

[6] Dykas MJ, Cassidy J. Attachment and the processing of social information across the lifespan: Theory and evidence. Psychol Bull. 2011; 137: 19–46. 10.1037/a0021367 21219056

[7] Waters TEA, Brockmeyer SL, Crowell JA. AAI coherence predicts caregiving and care seeking behavior: Secure base script knowledge helps explain why. Attach Hum Dev. 2013;15: 316–331. 10.1080/14616734.2013.782657 23566049

[8] Bosmans G, De Raedt R, Braet C. The invisible bonds: Does the secure base script of attachment influence children’s attention toward their mother? J Clin Child Adolesc Psychol. 2007; 36: 557–567. 18088214 10.1080/15374410701662717

[9] Bosmans G, Braet C, Koster E, De Raedt R. Attachment security is linked with attentional breadth in middle childhood. J Clin Child Adolesc Psychol. 2009; 38: 872–882. 10.1080/15374410903258926 20183670

[10] Ainsworth MDS. The development of infant-mother attachment In: Caldwell BM, Ricciuti HN, editors. Review of child development research. Vol. 3 Chicago (IL): University of Chicago; 1973 p. 1–94.

[11] Bowlby J. A secure base: Clinical applications of attachment theory. London: Rourledge; 1988.

[12] Kerns KA. Attachment in middle childhood In: Cassidy J, Shaver P, editors. Handbook of attachment. 2nd ed New York (NY): Guilford; 2008 p. 366–382.

[13] Mezulis AH, Hyde JS, Abramson LY. The developmental origins of cognitive vulnerability to depression: Temperament, parenting, and negative life events in childhood as contributors to negative cognitive style. Dev Psychol. 2006; 42: 1012–1025. 17087538 10.1037/0012-1649.42.6.1012

[14] Mogg K, Mathews A, Weinman J. Memory bias in clinical anxiety. J Abnorm Psychol. 1987; 96: 94–98. 3584672 10.1037//0021-843x.96.2.94

[15] MacLeod C, Cohen IL. Anxiety and the interpretation of ambiguity: A text comprehension study. J Abnorm Psychol. 1993; 102: 238–247. 8315136 10.1037//0021-843x.102.2.238

[16] Crick NR, Dodge KA. A review and reformulation of social information-processing mechanisms in children’s social-adjustment. Psychol Bull. 1994; 115: 74–101.

[17] MacLeod C, Mathews A, Tata P. Attentional bias in emotional disorders. J Abnorm Psychol. 1986; 95: 15–20. 3700842 10.1037//0021-843x.95.1.15

[18] Baert S, De Raedt R, Koster EHW. Modification of information-processing biases in emotional disorders: Clinically relevant developments in experimental psychopathology. Int J Cogn Ther. 2011; 4: 208–222.

[19] Bar-Haim Y, Lamy D, Pergamin L, Bakermans-Kranenburg M, Van Ijzendoorn M. Threat-related attentional bias in anxious and non-anxious individuals: A meta-analytic study. Psychol Bull. 2007; 133: 1–24. 17201568 10.1037/0033-2909.133.1.1

[20] Main M, Kaplan N, Cassidy J. Security in infancy, childhood and adulthood: A move to the level of representation. In: Bretherton I, Waters E, editors. Monogr Soc Res Child Dev. 1968; 50: 66–104.

[21] Kirsh SJ, Cassidy J. Preschoolers’ attention to and memory for attachment-relevant information. Child Dev. 1997; 68: 1143–1153. 9418230

[22] Belsky J, Spritz B, Crnic K. Infant attachment security and affective-cognitive information processing at age three. Psychol Sci. 1996; 2: 111–114.

[23] Miller EK, Cohen JD. An integrative theory of prefrontal cortex function. Annu Rev Neurosci. 2001; 24: 167–202. 11283309 10.1146/annurev.neuro.24.1.167

[24] Derryberry D, Tucker DM. Motivating the focus of attention In: Niedenthal PM, Kitayama S, editors. Heart’s eye: Emotional influences in perception and attention. New York (NY): Academic Press; 1994 p. 167–196.

[25] Kaplan RL, Van Damme I, Levine LJ. Motivation matters: Differing effects of pre-goal and post-goal emotions on attention and memory. Front Psychol. 2012; 3: 1–9. 10.3389/fpsyg.2012.00001 23162490 PMC3498897

[26] Easterbrook JA. The effect of emotion on cue utilization and the organisation of behaviour. Psychol Rev. 1959; 66: 183–201. 13658305 10.1037/h0047707

[27] Eysenck MW. Anxiety: The cognitive perspective. Hove: Lawrence Erlbaum Associates; 1992.

[28] Ridenour TA, Greenberg MT, Cook ET. Structure and validity of people in my life: self-report measure of attachment in late childhood. J Youth and Adolesc. 2006; 35: 1037–1053. 17476310 10.1007/s10964-006-9070-5 PMC1862408

[29] Maier MA, Bernier A, Pekrun R, Zimmermann P, Grossmann KE. Attachment working models as unconscious structures: An experimental test. International J of Behav Dev. 2004; 28: 180–189.

[30] Bosmans G, Koster E, Vandevivere E, Braet C, De Raedt R. Young adolescent’s confidence in maternal support: Attentional bias moderates the link between attachment-related expectations and behavioral problems. Cognit Ther Res. 2013; 37: 829–839.

[31] Easterbrooks MA, Davidson CE, Chazan R. Psychological risk, attachment, and behavior problems among school-aged children. Dev Psychopathol. 1993; 5: 389–402.

[32] Moss E, Cyr C, Dubois CK. Attachment at early school age and developmental risk: Examining family contexts and behavior problems of controlling-caregiving, controlling-punitive, and behaviorally disorganized children. Dev Psychol. 2006; 40: 519–532 10.1037/0012-1649.40.4.519 15238040

[33] Waters E, Sroufe LA. Social competence as a developmental construct. Dev Rev. 1983; 3: 79–97.

[34] Sroufe LA, Fox N, Pancake V. Attachment and dependency in developmental perspective. Child Dev. 1983; 55: 17–29.

[35] Crowell JA, Treboux D, Gao Y, Fyffe C, Pan H, Waters E. Assessing secure base behavior in adulthood: Development of a measure, links to adult attachment representations, and relations to couples’ communication and reports of relationships. Dev Psychol. 2002; 38: 679–693. 12220047

[36] Ammaniti M, Speranza AM, Fedele S. Attachment in infancy and in early and late childhood: A longitudinal study In: Kerns KA, Richardson RA, editors. Attachment in middle childhood. New York (NY): Guilford; 2005 p. 115–136.

[37] Bar-Haim Y, Sutton DB, Fox NA, Marvin RS. Stability and change of attachment at 14, 24, and 58 months of age: behavior, representation, and life events. J Child Psychol Psychiatry. 2000; 41: 381–388. 10784085

[38] Main M, Cassidy J. Categories of response to reunion with the parent at age 6: Predictable from infant attachment classifications and stable over a 1-month period. Dev Psychol. 1988; 24: 415–426.

[39] Shouldice A, Stevenson-Hinde J. Coping with security distress: The separation anxiety test and attachment classification at 4.5 years. J Child Psychol Psychiatry. 1992; 33: 331–348. 1564077 10.1111/j.1469-7610.1992.tb00870.x

[40] Slough NM, Greenberg MT. Five-year-olds’ representations of separation from parents: Responses from the perspective of self and other. New Dir Child Adolesc Dev. 1990; 48: 67–84. 10.1002/cd.23219904806 2216012

[41] Charlesworth L, Wood J, Viggiani P. Middle childhood In: Hutchison ED, editor. Dimensions of human behavior: The changing life course. New York (NY): Guilford; 2008 p. 177–226.

[42] Dwyer KM. The meaning and measurement of attachment in middle and late childhood. Hum Dev. 2005; 48: 155–182.

[43] Rutter M. Clinical implications of attachment concepts: retrospect and prospect. J Child Psychol Psychiatry. 1995; 126: 520–533. 10.1111/j.1469-7610.1995.tb02314.x 7650083

[44] Mayseless O. Ontogeny of attachment in middle childhood: Conceptualization of normative changes In: Kerns KA, Richardson RA, editors. Attachment in middle childhood. New York (NY): Guilford; 2005 p. 1–23.

[45] Gunnar MR, Talge NM, Herrera A. Stressor paradigms in developmental studies: What does and does not work to produce mean increases in salivary cortisol. Psychoneuroendocrinology. 2009; 34: 953–967. 10.1016/j.psyneuen.2009.02.010 19321267 PMC2692557

[46] Mogg K, Bradley BP, Williams R. Attentional bias in anxiety and depression: The role of awareness. Br J Clin Psychol. 1995; 34: 17–36. 7757037 10.1111/j.2044-8260.1995.tb01434.x

[47] Ball KK, Beard BL, Roenker DL, Miller RL, Griggs DS. Age and visual search: Expanding the useful field of view. J Opt Soc Am. 1988; 5: 2210–2219. 3230491 10.1364/josaa.5.002210

[48] Eldar S, Ricon T, Bar-Haim Y. Plasticity in attention: Implications for stress response in children. Behav Res Ther. 2008; 46: 450–461. 10.1016/j.brat.2008.01.012 18313034

[49] Armsden GC, Greenberg MT. The Inventory of Parent and Peer Attachment: Individual differences and their relationship to psychological well-being in adolescence. J Youth Adolesc. 1987; 16: 427–454. 10.1007/BF02202939 24277469

[50] Adams EK, Chase-Lansdale PL. Home sweet home: Parental separations, residential moves, and adjustment problems in low-income adolescent girls. Dev Psychol. 2002; 38: 792–805. 12220056

[51] Allen JP, Porter M, McFarland C, McElhaney KB, Marsh P. The relation of attachment security to adolescents’ paternal and peer relationships, depression and externalizing behaviour. Child Dev. 2007; 78: 1222–1239. 17650135 10.1111/j.1467-8624.2007.01062.x PMC2413435

[52] Belsky J, Jaffee S, Hsieh K, Silva P. Childrearing antecedents of intergenerational relations in young adulthood: A prospective study. Dev Psychol. 2001; 37: 801–814. 11699754 10.1037//0012-1649.37.6.801

[53] Spielberger CD, Edwards CD, Lushene R, Montuori J, Platzek D. State-Trait Anxiety Inventory for Children (manual). Palo Alto (CA): Consulting Psychologists Press; 1973.

[54] Bakker FC, van Wieriengen PCW, van der Ploeg HM, Spielberger CD. Handleiding bij de Zelf-Beoordelings-Vragenlijst voor Kinderen Een Nederlandstalige bewerking van de State-Trait Anxiety Inventory for Children. Lisse: Swets & Zeitlinger; 1989.

[55] Mesman J, Koot HM. Child-reported depression and anxiety in pre-adolescence: I. Associations with parent- and teacher-reported problems. J Am Acad Child Adolesc Psychiatry. 2000; 39: 1371–1378. 11068892 10.1097/00004583-200011000-00011

[56] Aiken LS, West SG. Multiple regression: Testing and interpreting interactions. Newbury Park (CA): Sage; 1991.

[57] Hayes AF, Matthes J. Computational procedures for probing interactions in OLS and logistic regression: SPSS and SAS implementations. Behav Res Methods. 2009; 41: 924–936. 10.3758/BRM.41.3.924 19587209

[58] Sroufe LA, Waters E. Attachment as an organizational construct. Child Dev. 1977; 48: 1184–1199.

[59] Gable P, Harmon-Jones E. The motivational dimensional model of affect: Implications for breadth of attention, memory, and cognitive categorization. Cogn Emot. 2010; 24: 322–337.

[60] Anderson CJ. The psychology of doing nothing: Forms of decision avoidance result from reason and emotion. Psychol Bull. 2003; 129: 139–167. 12555797 10.1037/0033-2909.129.1.139

[61] Cassidy J. Emotion regulation: Influences of attachment relationships. In: Fox NA, editor. Mon Soc Res Child Dev. 1994; 59: 228–249. 7984163

[62] Simpson JA, Rholes WS, Nelligan JS. Support seeking and support giving within couples in an anxiety-provoking situation: The role of attachment styles. J Pers Soc Psychol. 1992; 62: 434–446.

[63] Fraley RC. A connectionist approach to the organization and continuity of working models of attachment. J Pers. 2007; 75: 1157–1180. 17995461 10.1111/j.1467-6494.2007.00471.x

[64] Vandevivere E, Braet C, Bosmans G, Mueller S, De Raedt R. Attachment and children's biased attentional processing: Evidence for the exclusion of attachment-related information. PLoS One. 2014; 9: e103476–e103476. 10.1371/journal.pone.0103476 25061662 PMC4111605

[65] Ainsworth MDS. Attachments across the life span. Bull N Y Acad Med. 1985; 61: 792–812. 3864511 PMC1911889

[66] Kerns KA, Klepac L, Cole A. Peer relationships and preadolescents' perceptions of security in the child–mother relationship. Dev Psychol. 1996; 32: 457–466.

[67] Psouni E, Apetroaia A. Measuring scripted attachment-related knowledge in middle childhood: The secure base script test. Attach Hum Dev. 2014; 16: 22–41. 10.1080/14616734.2013.804329 23777439

[68] Dalgleish T, Taghavi R, Neshat-Doost H, Moradi A, Canterbury R, Yule W. Patterns of processing bias for emotional information across clinical disorders: A comparison of attention, memory, and prospective cognition in children and adolescents with depression, generalized anxiety, and posttraumatic stress disorder. J Clin Child Adolesc Psychol. 2003; 32: 10–21. 12573928 10.1207/S15374424JCCP3201_02

[69] Macleod C, Koster E, Fox E. Whither cognitive bias modification research? Commentary on the special section articles. J Abnorm Psychol. 2009; 24: 89–99. 10.1037/a0014878 19222317

